# Geo-epidemiology of autoantibodies in rheumatoid arthritis: comparison between four ethnically diverse populations

**DOI:** 10.1186/s13075-023-03009-7

**Published:** 2023-03-08

**Authors:** Emma C. de Moel, Leendert A. Trouw, Chikashi Terao, Nimmisha Govind, Mohammed Tikly, Hani El-Gabalawy, Irene Smolik, Holger Bang, Tom W. J. Huizinga, René E. M. Toes, Diane van der Woude

**Affiliations:** 1 Department of Rheumatology, Leiden University Medical Center, P.O. Box 9600, 2300 RC Leiden, the Netherlands; 2 Department of Genomic Medicine, Kyoto University Graduate School of Medicine, Kyoto, Japan; 3 Division of Rheumatology, University of the Witwatersrand, Johannesburg, South Africa; 4 Department of Internal Medicine, University of Manitoba, Winnipeg, Canada; 5 Orgentec Diagnostika GmbH, Mainz, Germany

**Keywords:** Rheumatoid arthritis, Autoantibodies, Epidemiology, Ethnicity, Genetics

## Abstract

**Background:**

Rheumatoid arthritis (RA) occurs across the globe in different ethnic populations. Most RA patients harbor anti-modified protein antibodies (AMPA); however, it is unclear whether differences exist in autoantibody responses at different geographic locations and between different ethnic groups, which could provide new clues regarding factors underlying autoantibody development. We therefore investigated AMPA prevalence and association with HLA DRB1 alleles and smoking in four ethnically diverse populations on four different continents.

**Methods:**

Anti-carbamylated (anti-CarP), anti-malondialdehyde acetaldehyde (anti-MAA), and anti-acetylated protein antibodies (anti-AcVim) IgG were determined in anti-citrullinated protein antibody-positive Dutch (NL, *n* = 103), Japanese (JP, *n* = 174), First Nations Peoples in Canada (FN, *n* = 100), and black South African (SA, *n* = 67) RA patients. Ethnicity-matched local healthy controls were used to calculate cut-offs. Risk factors associated with AMPA seropositivity in each cohort were identified using logistic regression.

**Results:**

Median AMPA levels were higher in First Nations Peoples in Canada and especially South African patients, as reflected by percentage seropositivity: NL, JP, FN, and SA: anti-CarP: 47%, 43%, 58%, and 76% (*p* < 0.001); anti-MAA: 29%, 22%, 29%, and 53% (*p* < 0.001); and anti-AcVim: 20%, 17%, 38%, and 28% (*p* < 0.001). Total IgG levels also differed markedly, and when autoantibody levels were normalized to total IgG, differences between cohorts became less pronounced. Although there were some associations with AMPA and HLA risk alleles and smoking, none was consistent across all four cohorts.

**Conclusions:**

AMPA against various post-translational modifications could consistently be detected on different continents across ethnically diverse RA populations. Differences in AMPA levels corresponded to differences in total serum IgG levels. This suggests that, despite differences in risk factors, a common pathway may be involved in AMPA development across geographic locations and ethnicities.

**Supplementary Information:**

The online version contains supplementary material available at 10.1186/s13075-023-03009-7.

## Introduction

Rheumatoid arthritis (RA) is a chronic autoimmune disease of unknown etiology that mainly affects the joints and is associated with circulating autoantibodies. Several RA-associated autoantibodies recognize protein epitopes that have been post-translationally modified, and are therefore known as anti-modified protein antibodies or AMPAs. The best-known post-translational modification (PTM) in RA is the enzymatic conversion of arginine to citrulline, which is recognized by anti-citrullinated protein antibodies or ACPA. More recently, other autoantibody systems have also gained attention, including anti-carbamylated protein antibodies (anti-CarP) recognizing homocitrulline-containing antigens, anti-acetylated peptide antibodies (AAPA) recognizing acetylated lysine, and anti-malondialdehyde-acetaldehyde antibodies (anti-MAA) recognizing proteins that are modified by adducts formed under oxidative stress [1–3].

The discovery of AMPA, and in particular ACPA, has had a great impact on current pathophysiological hypotheses regarding RA, due to ACPA’s striking association with classical RA risk factors. The most important genetic risk factor for RA, the HLA shared epitope (SE) alleles (or the amino acids in distinct HLA DBR1 positions of these alleles), is now known to be primarily associated with the ACPA-positive subset of disease [4]. This has given rise to hypotheses of different pathophysiological mechanisms underlying ACPA-positive versus ACPA-negative RA. Although a similar predilection for ACPA-positive RA has also been described with regard to smoking, more recent reports support the view that smoking is more strongly associated with rheumatoid factor and concurrent presence of multiple antibodies rather than ACPA [5–8].

Despite these insights, the exact etiological pathways leading to autoantibody formation are still unclear. It is conceivable that environmental factors may increase the risk for the development of autoantibodies in a geographic or population-specific manner, as is the case in other autoimmune diseases. For example, research in a specific population of Amerindians in Brazil has led to remarkable insights in fogo selvagem, a blistering autoimmune dermatological disease with resemblance to pemphigus. In this population, a high percentage of healthy individuals have low levels of anti-desmoglein 1 antibodies which has been found to be strongly associated with infestation with black flies. Only in individuals carrying HLA susceptibility alleles does epitope spreading occur, leading to pathogenic antibodies directed against particular subdomains of desmoglein 1 [9]. Because black flies occur only endemically and only induce disease in genetically susceptible locals, this disease is an example of an exceptional interplay between population-specific environmental and genetic risk factors. In this way, a rare environmental factor may be intimately involved in disease initiation and can possibly clarify underlying pathophysiological mechanisms of disease.

This example illustrates that investigating autoantibody responses in diverse locations and ethnic groups can lead to the elucidation of novel factors playing a role in the development of the autoantibody response and of the resulting disease. RA occurs in many ethnically diverse populations around the world, with different genetic frameworks and environmental exposures. Therefore, we set out to characterize the AMPA response in four ethnically diverse RA populations originating from four different continents, since differences in autoantibody presence could point to novel risk factors and shed more light on the development of AMPA in RA.

## Methods

### Study population

The study population consisted of four cohorts of patients fulfilling the 1987 revised ACR criteria for RA [10]. It was not possible to obtain information on the fulfillment of the 2010 RA criteria for all cohorts. All patients supplied informed consent, and study protocols were approved by the relevant local ethics committee.

The Dutch RA patients (NL) were part of the Early Arthritis Cohort (EAC), a prospective cohort initiated in 1993 at the Leiden University Medical Center (LUMC) [11, 12]. Patients were of white European ancestry. RA was diagnosed at 1-year follow-up and sera used in this study were collected at this time point. Healthy controls were recruited from the Leiden area.

The Canadian patients and controls came from an Indigenous North American population in Manitoba: First Nations Peoples in Canada (FN), who have an unusually high prevalence of RA [13, 14]. All patients and controls were of self-reported Cree and Ojibway descent. Serum was collected either at the baseline visit or at a subsequent visit.

In Japan (JP), RA patients and healthy controls of Japanese descent were recruited in a cross-sectional manner from 5 hospitals in the Kyoto University area [15].

The South African population (SA) consisted of black RA patients with less than 2 years of disease duration recruited from two tertiary hospitals in South Africa participating in the Gauteng Rheumatoid Evaluation Assessment Trial (GREAT) [16]. South African controls were healthy black laboratory and clinical personnel at the University of Witwatersrand.

### Clinical data

All included patients were ACPA-positive, determined by clinical second- and third-generation anti-CCP enzyme-linked immunosorbent assays (ELISAs) [11, 12, 14–16].

For the Dutch cohort, smoking habits were recorded at the time of sera collection (or at baseline if 1-year data was not available) by a trained research nurse or physician. For the Canadian cohort, patients filled in an extended smoking history questionnaire at inclusion. For the South African and Japanese cohorts, smoking history (ever or never) was recorded at inclusion.

HLA four-digit genotyping data was available for most patients of each cohort. The SE alleles were defined as described previously [4]. Patients were considered SE or HLA-DRB1*03-positive (which may be related to anti-CarP positivity) if they were homo- or heterozygous for these alleles.

### Detection of serum autoantibodies by ELISA

By ELISA, total IgG and four AMPAs were tested: anti-CarP fetal calf serum (FCS) IgG, anti-malondialdehyde acetaldehyde (MAA) FCS IgG, anti-acetylated-lysine vimentin IgG (anti-AcVim of the AAPA family), and anti-CCP2 IgG.

Total IgG levels were determined using Bethyl Laboratories reagents and protocol (Bethyl Labs E80-104). In-house ELISAs were conducted essentially as previously described for anti-CarP FCS IgG [1] and anti-CCP2 IgG [17]. Anti-AcVim IgG was tested using an OrgenTec kit (Orgentec Diagnostika GmbH, Germany) [3]. Anti-MAA FCS IgG used the same in-house protocol as anti-CarP IgG except coating with MAA-FCS and sham-FCS protein, serum dilutions at 1:1000, and detection by rabbit anti-human IgG-HRP antibody. MAA-FCS and sham-FCS were prepared as follows: a solution of 0.5 M tetramethoxypropane and 0.3% hydrochloric acid was incubated at 37 °C for 12 min with agitation. A new solution of 20% of the above, 4% of acetaldehyde, and 1–5 mg/ml FCS was prepared. pH was brought to 4.8, and this solution and sham-FCS were incubated at 37 °C for 2 h. Finally, both samples were dialyzed for 32 h against phosphate-buffered saline, refreshing multiple times, yielding MAA-FCS and sham-FCS at around 4 mg/mL.

For all AMPA ELISAs, absorbance values were converted to arbitrary units per milliliter (aU/mL) using a titration curve of pooled, serially diluted Dutch patient sera positive for that AMPA. We established cohort-specific cut-offs using each cohort’s respective healthy controls’ mean aU/mL plus two standard deviations, resulting in four distinct cut-offs (Supplementary Fig. 1). Additionally, for a patient sample to be considered positive, the specific OD on the modified peptide had to be more than 0.1 optical density (OD) above the signal on the non-modified peptide.

### Statistical analysis

Chi-squared tests and Kruskal–Wallis tests were used to examine differences between cohorts in the prevalence and levels of AMPAs, as well as cohort characteristics. Logistic regression was used to identify whether smoking was associated with AMPA seropositivity in each cohort, corrected for gender. Logistic regression was also used to examine the association of HLA DRB1 alleles with AMPA seropositivity. Analyses were performed with Stata 14.1: Special Edition (StataCorp LP, TX, USA).

## Results

### Cohort characteristics

The cohort characteristics of the four geographically and ethnically diverse ACPA-positive RA populations are summarized in Table 1.

**Table 1:** Cohort characteristics

	Netherlands *N* = 103	First Nations (Canada) *N* = 100	Japan *N* = 174	South-Africa *N* = 67	*p*-value
Sex, female	66%	79%	82%	89%^1^	**0.002**
Age, median years (IQR)	58 (49–65)	48 (35–55)	60 (48–67)	49 (41–56)^7^	**< 0.001**
Disease duration, median years (IQR)	1.3 (1.1–1.8)^1^	7.7 (2.5–17.0)	7.5 (4.8–14.8)^1^	0 (0.0–0.0)^1^	**< 0.001**
RF positive	90%	93%^1^	95%	100%	0.06
Ever smokers	60%^5^	84%	28%^4^	12%	**< 0.001**
HLA SE present	85%	94%^37^	78%	71%^1^	**< 0.001**
HLA-DRB1*03 present	14%	5%^37^	0%	32%^1^	**< 0.001**
Anti-CarP positive	50%	60%	45%	79%	**< 0.001**
Anti-MAA positive	29%	29%^2^	22%^2^	53%^1^	**< 0.001**
Anti-AcVim positive	23%^1^	40%^6^	22%^30^	46%^6^	**< 0.001**
Treatment at time of sample draw	*N* data = 72	*N* data = 72	*N* data = 174	*N* data = 67	
DMARD-naïve	0 (0%)	6 (8%)	0 (0%)	58 (86%)	
Not DMARD-naïve	72 (100%)	66 (92%)	174 (100%)	9 (14%)	

Superscripted numbers indicate the number of missing data per characteristic per cohort. Significance is based on Pearson’s chi-square test, *t*-test, or Kruskal–Wallis test, as appropriate. Disease duration refers to the time in years between the date of 1st presentation and the date of the sample draw. For South Africa, only 9 patients had samples drawn at 6 months and the rest at baseline. Symptom duration refers to the time in years between the date of symptoms and the date of the sample draw. For DMARD use, categories overlap for patients using multiple agents. Other DMARDs include azathioprine, sulfasalazine, hydroxychloroquine, leflunomide, cyclosporine, bucillamine, gold, and minocycline

*p*-values printed in bold were statistically significant

Cohorts differed significantly for all baseline characteristics except RF status. Notably, patients in the FN and SA cohorts were younger and the NL and SA cohorts had shorter disease durations, while the JP and SA cohorts had few smokers.

### Autoantibody levels and prevalence in ethnically diverse RA populations

**Fig. 1:**
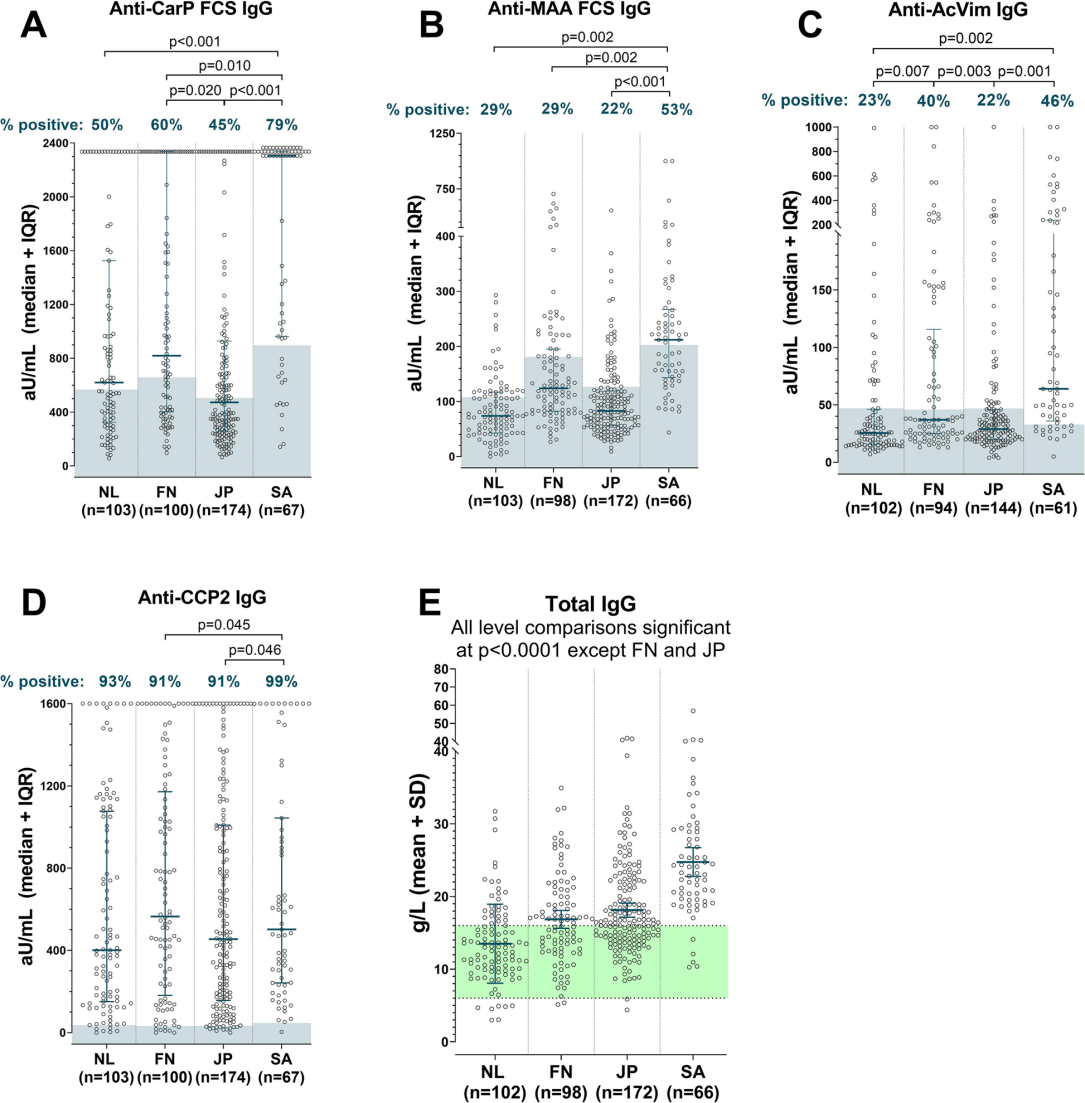
AMPA levels in geographically and ethnically diverse RA populations. Levels in arbitrary units (aU/mL) for four AMPAs and grams per liter (g/L) for total IgG in the serum of four geographically and ethnically diverse RA populations. Patients clustered at the maximum were above the highest standard of the ELISA. Blue shading indicates the patients falling below the cohort-specific positivity cut-off (see Supplementary Fig. 1 for data regarding cut-off determination in healthy controls and reactivity to the control peptide); green shading is the normal range for total IgG (established in European cohorts). Lines indicate the median and interquartile range. *p*-values correspond to chi-square tests on the proportion of patients considered positive, indicated in percentages above graphs

Figure 1 displays the levels of the AMPAs in all four cohorts.

Remarkable differences were seen between the cohorts with levels generally being higher in FN and SA RA patients. This pattern of higher reactivity against the PTM antigens could also be seen to a lesser extent in the healthy controls of these two cohorts, although in each population, responses of healthy controls were clearly lower than those of RA patients (Supplementary Fig. 1). Reactivity against the non-modified control antigens was lower than the PTM antigens in all cohorts. Although South African RA patients had higher anti-lysine and anti-arginine reactivity as compared to RA patients from other cohorts, their reactivity to the corresponding PTM antigen was also higher, leading to a high prevalence of PTM-specific reactivity.

Despite the differences in cohort-specific cut-offs, the higher levels in the FN and SA RA patients also translated into a higher prevalence of several AMPA in these cohorts (Fig. 1 and Table 1). Most notably, anti-CarP and anti-AcVim antibodies were more prevalent in FN and SA patients.

The level differences between the cohorts remained when levels were compared only in patients that were positive for AMPA (Supplementary Fig. 2).

Anti-CCP2 IgG was retested in these ACPA-positive patients to determine levels. Retest positivity for anti-CCP2 IgG was 93% on average (Fig. 1D) with the most discrepancy found in patients first tested by anti-CCP3 test (not shown). When analyses were performed only in the patients who retested positive for anti-CCP2 IgG (as a sensitivity analysis), the results did not differ.

### AMPA levels in relation to total IgG

**Fig. 2:**
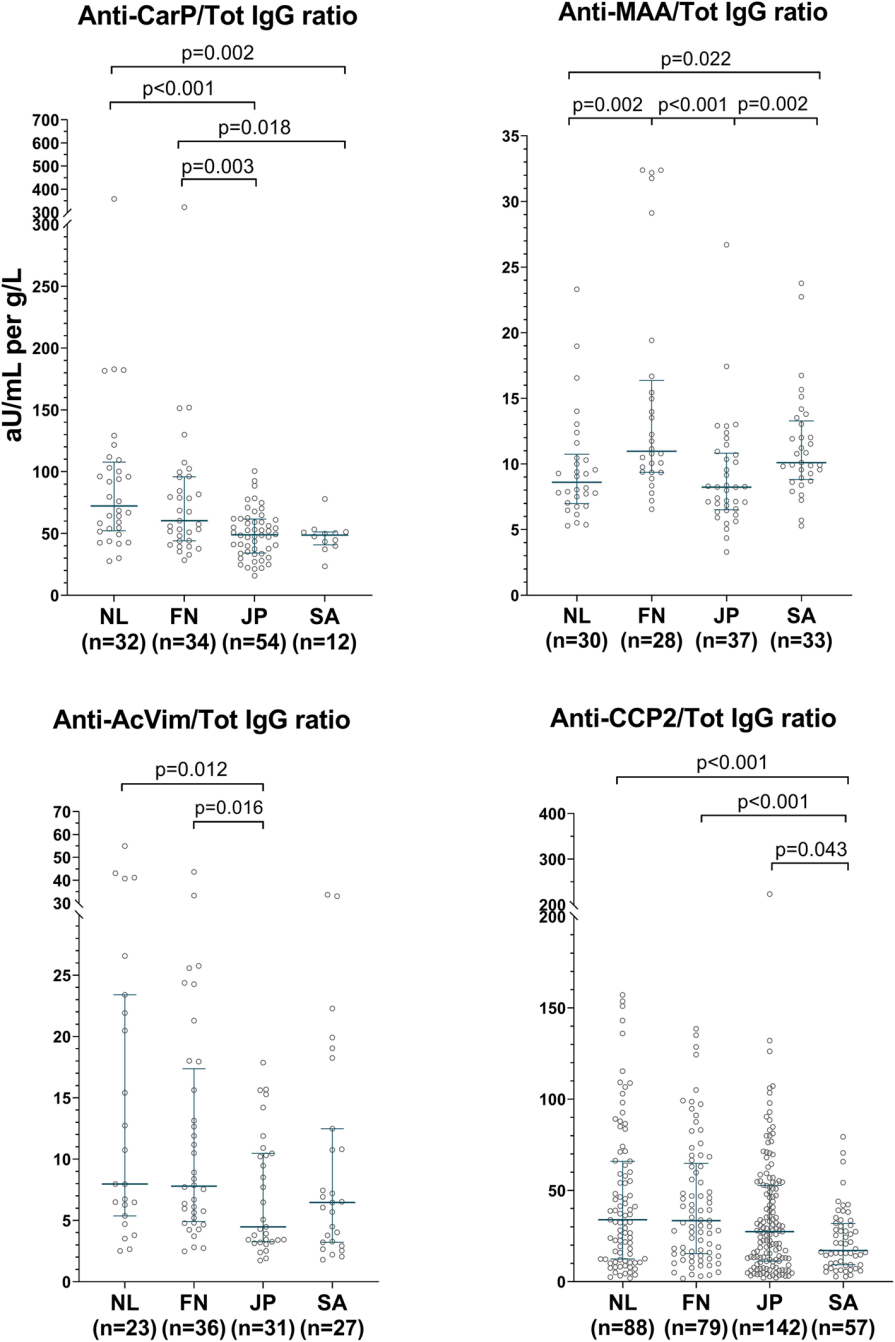
AMPA to IgG ratios in geographically and ethnically diverse RA populations. Ratios of levels in arbitrary units (aU/mL) for four AMPAs, per grams per liter (g/L) total IgG in the serum of four RA populations. Ratios are only shown in patients that were positive for the AMPA. Lines indicate median and interquartile range; *p*-values correspond to Mann–Whitney *U* tests. The range of aU/mL per g/L is not directly comparable between AMPAs

Various studies suggest that differences in total IgG levels may follow ethnographic lines [18–20]. To account for the possibility that the AMPA differences correspond to total IgG differences, we also measured total IgG. Levels of total IgG to some extent resembled AMPA levels in that SA patients had the highest levels of both (Fig. 1). Next, we calculated ratios of AMPA levels divided by IgG levels (Fig. 2).

Although some of these ratios still differed significantly between cohorts, overall differences were now less striking, suggesting that autoantibody-level differences may largely correspond to cohort-specific differences in total IgG.

When patients with levels above the highest standard of an ELISA were removed from the analysis, this did not greatly change the results (Supplementary Fig. 3).

### Association of AMPA positivity and smoking, HLA DRB1*03, and SE

**Fig. 3:**
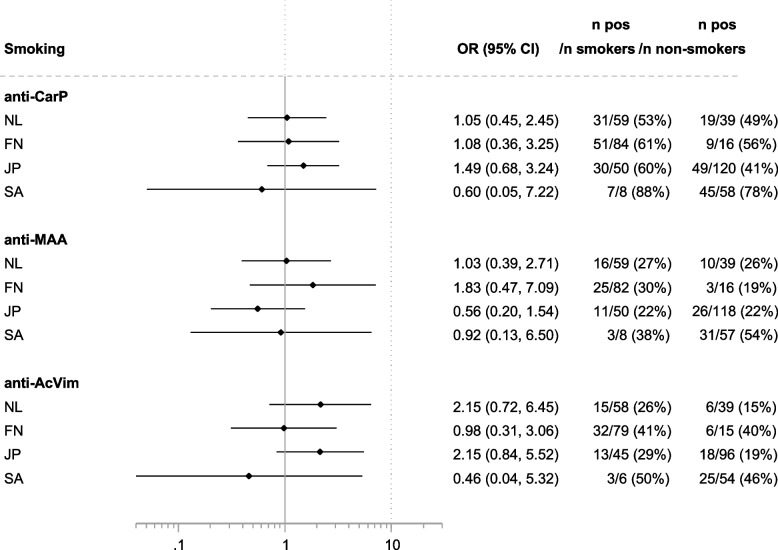
Association of the different AMPA with smoking in the four populations. The association with smoking was corrected for gender. Please note that in South Africa, correction for gender had a large effect on odds ratios because very few women smoke

**Fig. 4:**
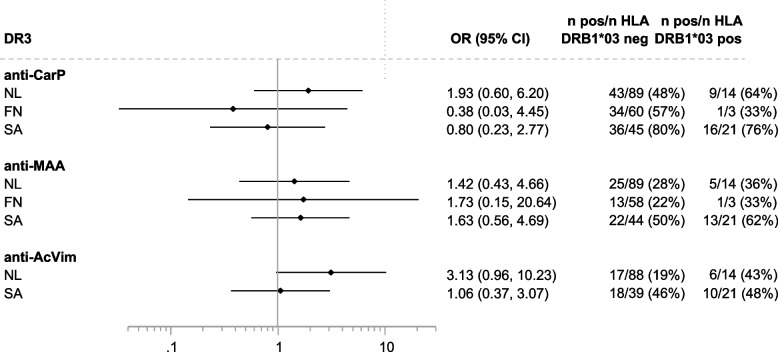
Associations of the different AMPA with the presence of HLA DRB1*03 alleles. Genetic associations were not corrected for gender

**Fig. 5:**
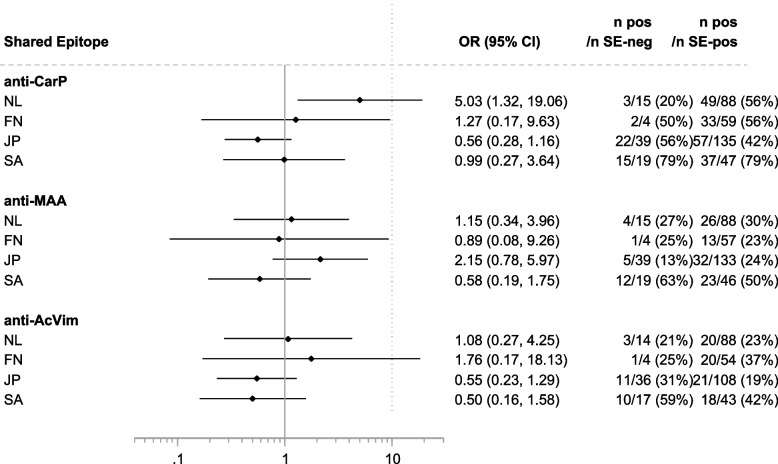
Associations of the different AMPA with the presence of shared epitope alleles. Genetic associations were not corrected for gender

Next, we aimed to delineate whether the presence of a certain AMPA might be associated with the known risk factors for autoantibody-positive RA (smoking and HLA risk alleles). To avoid finding spurious associations, we limited our analyses to HLA SE and DRB1*03 alleles. Since the levels of AMPA and the cut-offs differed per cohort (as described above), all association analyses were performed based on dichotomous seropositive versus seronegative autoantibody results, rather than autoantibody levels. The results are displayed in Figs. 3, 4, and 5.

Regarding smoking, there was no association with anti-CarP or anti-MAA. However, there appeared to be a positive association with smoking and anti-AcVim in NL and JP, although this did not achieve statistical significance.

For HLA DRB1*03, which was not present in JP and only rarely in FN, there were several positive albeit non-significant associations with the different AMPA. This was found for anti-CarP in NL (which has been reported before in ACPA-negative patients [21]) and for anti-MAA in several cohorts and anti-AcVim in NL.

With regard to the HLA SE alleles, there was a positive association with anti-CarP in NL. For the other cohorts, there were no associations with anti-CarP, anti-MAA, or anti-AcVim in these ACPA-positive patients.

## Discussion

In light of the recent discovery of several different RA-associated autoantibodies directed against post-translational modifications (AMPA), we determined three major novel AMPA in four geographically and ethnically diverse cohorts of ACPA-positive RA patients, to elucidate whether there might be population-specific risk factors involved in their development. We found differences between cohorts in the prevalence and median levels of anti-CarP, anti-MAA, and anti-AcVim. However, this diversity corresponded to a large extent to cohort-specific variation in total IgG. Finally, there were no associations between AMPA and the classic risk factors HLA SE, HLA DRB1*03, or smoking that were consistent across all cohorts.

Our results are in line with a limited number of previous publications investigating the prevalence of ACPA and anti-CarP in different ethnic populations and geographic locations. A study from Malaysia investigating RA patients of three different Asian backgrounds (Malay, Chinese, and Indian) reported similar frequencies of ACPA among these patients and an association with HLA SE alleles [22]. Likewise, studies from Africa have also found relatively similar ACPA prevalences [16, 23]. Regarding the presence of anti-CarP, the previously reported estimates in the same Indigenous North American population and in a similar Japanese cohort are fairly in line with our observations [24, 25]. However, to the best of our knowledge, our current study is the first to measure AAPA and anti-MAA in RA patients living outside Europe or the USA and to provide a direct comparison of AMPA measured with the same methods in 4 ethnically diverse RA populations from 4 different continents. Our study leads to new important insights regarding the higher seroprevalence of autoantibodies in populations characterized by high total IgG levels. This is a very relevant finding for future studies focusing on autoantibodies in African populations, which are generally underrepresented in medical research.

The higher AMPA and IgG levels found in black South African RA patients may be due to several independent factors. First, there are multiple publications describing higher IgG levels in people of African descent although the mechanisms underlying this intriguing observation remain unclear [19, 20]. While environmental factors such as diet or parasitic infections have been suggested, higher IgG levels in black West Africans residing in Britain for several years have also pointed towards genetic factors [26]. Furthermore, treatment of RA is known to be associated with a decrease in AMPA levels [27], and in contrast to the other cohorts, the South African patients were largely treatment-naïve at the moment of serum sampling.

We found no consistent associations of smoking and SE with AMPA positivity in the investigated cohorts. At the most, there was a positive associative trend for smoking and AAPA in NL and JP, but not in the others. This lack of association could have been due to the limited size of the cohorts, although the effect sizes generally did not indicate that greater power would have resulted in significant findings. The positive association of SE with anti-CarP in NL is surprising considering that this has not been found before in analyses of larger datasets [28, 29]. This raises the question of whether the fact that the current analysis was performed in ACPA-positive patients only might have resulted in a spurious finding in one single cohort in this case.

Overall, our finding that the various AMPA could be detected in substantial amounts in RA patients of 4 different ethnicities on different continents implies that population-specific ethnic or geographical risk factors do not seem to play a major role in shaping the AMPA response once ACPA have developed. Contrary to the example of fogo sevalgem in which endemic black flies are a specific factor influencing autoantibody reactivity, this kind of effect does not appear to exist within autoantibody-positive RA. This indicates that the development of a broad AMPA response to different PTMs represents a final common outcome of different pathways leading to the development of ACPA and eventually RA. This hypothesis is in line with murine studies which have shown that immunization with a protein containing one particular PTM can induce cross-reactive AMPA against other PTMs as well [30]. However, cross-reactivity between different AMPA cannot be the sole explanation for our findings, considering that (1) both AAPA and anti-CarP-responses have been found to be only partly cross-reactive with citrullinated antigens [3, 31] and (2) anti-MAA-antibodies have been described to display no cross-reactivity at all [32]. Thus, it appears likely that other factors, apart from cross-reactivity, also play a role in shaping the AMPA response, and these processes, possibly involving somatic hypermutation of B cell receptors, or survival signals for AMPA-specific B cells, follow a common path in patients with different ethnic and geographical backgrounds.

The main limitation of this study is the fact that we had access only to ACPA-positive RA patients. This precludes any conclusions about the prevalence of each AMPA in the entire RA population consisting of both seropositive and seronegative patients. However, because non-ACPA AMPA occur largely in ACPA-positive RA, this selection does maximize our ability to identify AMPA-positive patients and compare AMPA levels. In addition, the presence of ACPA in all patients does make the comparison between cohorts more straightforward, since differences in ACPA presence would have been a major confounding factor. However, in light of the strong association between HLA risk factors and ACPA, the results regarding genetic associations should be interpreted with extreme caution. Another limitation is the differences in cohort demographics. We attempted to account for these differences by correcting our smoking analyses for gender, which most closely determined smoking habits. Disease duration also differed, which can be seen as a proxy for treatment effects; however, correction for this made some models statistically nonconvergent and did not change the results of the others. Data on clinical characteristics were limited, making it impossible to correlate observed differences in autoantibodies with disease activity, DMARD exposure, and clinical outcomes. Since information on other (environmental) exposures like previous infections, air pollution, or comorbidities was unavailable, we limited our analyses to the communal risk factors which were known for each cohort: smoking and HLA DRB1 alleles. A further limitation is the relatively small sample sizes in each cohort.

Strengths of our study include its innovative design in measuring several different AMPA in 4 different ethnic and geographical cohorts. This leads to relevant insights regarding differences not only in the levels of autoantibodies, but also of total IgG; a fact that has thus far attracted little attention in the rheumatology autoantibody literature. Another strength of the study is the fact that all AMPA determinations were performed in the same manner. All cohorts were analyzed in the same center on the same day and were represented on each ELISA plate, and the cut-offs were determined using cohort- and ethnicity-matched healthy controls. All these measures serve to maximize comparability between cohorts, which is not a trivial issue as illustrated by the fact that our data show clear differences in calculated cut-offs, thereby underlining the importance of using locally matched controls in determining cut-offs.

## Conclusion

In these geographically and ethnically diverse ACPA-positive RA populations, AMPA against other post-translational modifications were consistently present, with higher levels in the South African patients. However, total IgG levels were also higher in South African patients, and after correcting for this, differences were less striking. This suggests that in ACPA-positive patients with different genetic and environmental exposures, the development of a broad AMPA response to different PTMs may represent a final common outcome of autoimmune pathophysiology.

## Supplementary Information

**Additional file 1:** **Supplementary Figure 1.** AMPA and control peptide reactivity in RA and HCs. Levels in arbitrary units (aU/mL) for four AMPAs in the serum of four ethnically diverse RA populations and their ethnicity-matched healthy controls (HC). Grey dots indicate reactivity to the control peptide that was tested in tandem with the AMPA (which are shown with black dots). Blue violin plots display the median, interquartile range, and distribution of levels.NL: Netherlands, FN: First Nation, JP: Japan, SA: South Africa, RA: RA patients, HC: healthy controls. **Supplementary Figure 2. **Autoantibody levels in patients seropositive for each AMPAs in ethnically diverse RA populations. Levels in arbitrary units (aU/mL) for four AMPAs in the serum of four ethnically diverse RA populations. Patients clustered at the maximum were above the highest standard of the ELISA. Grey shading indicates the cohort-specific cut-off (see Supplementary Figure 2 for data regarding cut-off determination in healthy controls and reactivity to the control peptide). Lines indicate median and interquartile range and p-values correspond to Mann-Whitney U tests. **Supplementary Figure 3. **Autoantibody levels in patients seropositive for each AMPAs in ethnically diverse RA populations. Ratios of levels in arbitrary units (aU/mL) for four AMPAs per grams per liter (g/L) total IgG in the serum of four RA populations. Ratios are only shown in patients that were positive for the AMPA and that were also not above the highest standard. Lines indicate median and interquartile range; p-values correspond to Mann-Whitney U tests. The range of aU/mL per g/L are not directly comparable between AMPAs.

## Data Availability

The dataset used and/or analyzed during the current study are available from the corresponding author available on request, provided that permission has been obtained from the principal investigator of the respective cohort(s).

## Abbreviations

AAPA: Anti-acetylated peptide antibodies
ACPA: Anti-citrullinated protein antibodies
ACR: American College of Rheumatology
AMPA: Anti-modified protein antibodies
anti-AcVim: Anti-acetylated-lysine vimentin
anti-CarP: Anti-carbamylated protein antibodies
anti-CCP: Anti-cyclic citrullinated peptide
anti-MAA: Anti-malondialdehyde-acetaldehyde antibodies
aU/ml: Arbitrary units per milliliter
DMARD: Disease-modifying anti-rheumatic drug
EAC: Early Arthritis Clinic
ELISA: Enzyme-linked immunosorbent assay
FCS: Fetal calf serum
FN: First Nations (Peoples in Canada)
GREAT: Gauteng Rheumatoid Evaluation Assessment Trial
HLA: Human leukocyte antigen
HRP: Horseradish peroxidase
IgG: Immunoglobulin G
JP: Japan
LUMC: Leiden University Medical Center
NL: The Netherlands
OD: Optical density
PTM: Post-translational modification
RA: Rheumatoid arthritis
SA: South Africa
SE: Shared epitope
