# Supplementary material for: Geo-epidemiology of autoantibodies in rheumatoid arthritis: comparison between four ethnically diverse populations
Source: Arthritis Res Ther. 2023 Mar 8;25:37. doi: 10.1186/s13075-023-03009-7 (PMC9993621; doi:10.1186/s13075-023-03009-7)
Supplement: Supplementary file 1 — Additional file 1: Supplementary Figure 1. AMPA and control peptide reactivity in RA and HCs. Levels in arbitrary units (aU/mL) for four AMPAs in the serum of four ethnically diverse RA populations and their ethnicity-matched healthy controls (HC). Grey dots indicate reactivity to the control peptide that was tested in tandem with the AMPA (which are shown with black dots). Blue violin plots display the median, interquartile range, and distribution of levels.NL: Netherlands, FN: First Nation, JP: Japan, SA: South Africa, RA: RA patients, HC: healthy controls. Supplementary Figure 2. Autoantibody levels in patients seropositive for each AMPAs in ethnically diverse RA populations. Levels in arbitrary units (aU/mL) for four AMPAs in the serum of four ethnically diverse RA populations. Patients clustered at the maximum were above the highest standard of the ELISA. Grey shading indicates the cohort-specific cut-off (see Supplementary Figure 2 for data regarding cut-off determination in healthy controls and reactivity to the control peptide). Lines indicate median and interquartile range and p-values correspond to Mann-Whitney U tests. Supplementary Figure 3. Autoantibody levels in patients seropositive for each AMPAs in ethnically diverse RA populations. Ratios of levels in arbitrary units (aU/mL) for four AMPAs per grams per liter (g/L) total IgG in the serum of four RA populations. Ratios are only shown in patients that were positive for the AMPA and that were also not above the highest standard. Lines indicate median and interquartile range; p-values correspond to Mann-Whitney U tests. The range of aU/mL per g/L are not directly comparable between AMPAs. [file 13075_2023_3009_MOESM1_ESM.docx]

| **Supplementary Figure 1:** **AMPA and control peptide reactivity in RA and HCs**  Levels in arbitrary units (aU/mL) for four AMPAs in the serum of four ethnically diverse RA populations and their ethnicity-matched healthy controls (HC). Grey dots indicate reactivity to the control peptide that was tested in tandem with the AMPA (which are shown with black dots). Blue violin plots display the median, interquartile range, and distribution of levels.NL: Netherlands, FN: First Nation, JP: Japan, SA: South Africa, RA: RA patients, HC: healthy controls. |
| --- |
|        |

| **Supplementary Figure 2: Autoantibody levels in patients seropositive for each AMPAs in ethnically diverse RA populations**  Levels in arbitrary units (aU/mL) for four AMPAs in the serum of four ethnically diverse RA populations. Patients clustered at the maximum were above the highest standard of the ELISA. Grey shading indicates the cohort-specific cut-off (see **Supplementary Figure 2** for data regarding cut-off determination in healthy controls and reactivity to the control peptide). Lines indicate median and interquartile range and p-values correspond to Mann-Whitney U tests. |
| --- |
|  |

| **Supplementary Figure 3: Autoantibody levels in patients seropositive for each AMPAs in ethnically diverse RA populations**  Ratios of levels in arbitrary units (aU/mL) for four AMPAs per grams per liter (g/L) total IgG in the serum of four RA populations. Ratios are only shown in patients that were positive for the AMPA and that were also not above the highest standard. Lines indicate median and interquartile range; p-values correspond to Mann-Whitney U tests. The range of aU/mL per g/L are not directly comparable between AMPAs. |
| --- |
|  |
